# Non-Arteritic Anterior Ischemic Optic Neuropathy Associated With the Use of Phosphodiesterase Type 5 Inhibitors: A Literature Review

**DOI:** 10.7759/cureus.27642

**Published:** 2022-08-03

**Authors:** Mosab Hor, Ahmed M Baradeiya, Hodan Qasim, Mohamed Nasr, Amad Mohammad

**Affiliations:** 1 Ophthalmology, Children Retina Institute, Los Angeles, USA; 2 Ophthalmology, Palestinian Medical Council, Ramallah, PSE; 3 Research, Fresno Clinical Research Center, Fresno, USA; 4 Internal Medicine, Alfaisal University College of Medicine, Riyadh, SAU; 5 General Internal Medicine, El Mansoura General Hospital, Mansoura, EGY; 6 Hematology and Oncology, Saint James School of Medicine, Arnos Vale, VCT

**Keywords:** viagra, adverse effects, sexual dysfunction, erectile dysfunction, sildenafil, phosphodiesterase type 5 inhibitors, naion, non-arteritic anterior ischemic optic neuropathy, review, association

## Abstract

Phosphodiesterase type 5 (PDE5) inhibitors are frequently used for erectile dysfunction (ED) as the first line of treatment. This medication was initially developed to treat muscle spasms and pulmonary hypertension. The United States Food and Drug Administration (FDA) approved its usage for treating ED. Sildenafil, tadalafil, vardenafil, and avanafil are PDE5 inhibitors. The decrease of cyclic guanosine monophosphate (cGMP) in smooth muscle cells caused by sildenafil causes smooth muscle relaxation and penile erection. Vasodilation of the blood vessels reduces perfusion and blood flow to the optic nerve and eye. Several incidences of non-arteritic anterior ischemic optic neuropathy (NAION) have been recorded in sildenafil users, among other ocular complications. The onset of NAION is usually sudden and painless, and it is associated with any pattern of visual field loss. Possible symptoms include poor visual acuity, diminished color vision, a visual field defect, or hemorrhages in the form of flames. Nevertheless, NAION pathogenesis is still a mystery. Most visual effects are reversible weeks after the medication is stopped, and NAION does not seem to cause a permanent blindness. A small cup-to-disc ratio (disc at risk) and underlying systemic illnesses, such as hypertension, increase the risk of developing NAION. An early indicator of cardiovascular disease is ED. NAION diagnosis is challenging due to a lack of confirmatory diagnostic evidences. Normal visual acuity does not exclude NAION from being a possibility. In order to evaluate visual outcomes in NAION, data on both visual acuity (VA) and the full peripheral visual field are needed. Treatment with steroids did not seem to improve visual results.

## Introduction and background

An individual's inability to achieve and or maintain an erection for satisfactory sexual relations is referred to as erectile dysfunction (ED). It is a common condition, with over 18 million men affected worldwide [1]. Approximately 1-10% of males under the age of 40 suffer from ED. For men over 60, the prevalence rate rises to 20-100% [2]. As the first line of treatment, phosphodiesterase type 5 (PDE5) inhibitors are commonly prescribed for ED. This drug was initially developed to treat pulmonary arterial hypertension and muscle spasms [1]. ED is strongly associated with older age, though other lifestyle factors are also implicated, like smoking, drinking, inactivity, obesity, and sleep disorders [2]. The United States Food and Drug Administration (FDA) approved its use for treating ED in 1998 after clinical studies demonstrated its efficacy and safety [1].

Sildenafil (Viagra; Pfizer Inc., New York City, New York, USA) inhibits PDE5 and PDE6 (10-fold less potently than PDE5), as well as PDE24 and PDE711 isoenzymes. Reduced levels of cyclic guanosine monophosphate (cGMP) in smooth muscle cells, their relaxation, and penile erection are the outcomes of the inhibitory process [3]. The PDE5 inhibitors enzyme can cross the blood-brain and blood-retinal barriers, and it may partially inhibit PDE6 in the retina, affecting photoreceptors and the optic nerve in significant dose-dependent ways [1]. Around 10 mL/min/kg is the typical plasma clearance rate for sildenafil. The drug is primarily metabolized by the liver (cytochrome P4503A4) with a half-life of approximately three to five hours [3]. Physiological effects of PDE5 inhibition include vasodilation, which results in reduced blood flow to the eye and reduced perfusion of the optic nerve [4]. PDE6 is expressed in the retina, where it modifies the concentrations of cGMP and visual signal transduction. PDE5 is 700 times more sensitive to the effects of tadalafil than PDE6 and other PDEs. As a result, visual disturbances (including changes in color vision, light perception, blurred vision, and photophobia) are minimized compared to sildenafil. PDE5 is most strongly inhibited by vardenafil, while other PDEs are minimally affected [3]. Additionally, the widening of blood vessels in the lungs decreases pulmonary blood pressure (BP) and improves the function of the heart, which is effective in reducing pulmonary arterial hypertension [5]. The suggested dose of sildenafil is 25-50 mg; however, it can be raised to 100 mg three times a week. Adverse effects are more frequent in doses >100 mg, yet they do not lead to more efficacy [3].

Ocular problems include branch retinal artery blockage, angle-closure glaucoma, anterior ischemic optic neuropathy (AION), posterior ischemic optic neuropathy (PION), color vision impairment, optic nerve atrophy, and central serous chorioretinopathy [4]. Darker than usual colors and colored tinges (typically blue or green) on objects might be signs of a color vision impairment. In patients receiving sildenafil, bluish tinges may be caused by the selective nature of this drug to S cones (one of the subtypes of retinal cones that is responsible for short wavelength light sensing). These symptoms typically emerge one to two hours after the medications are administered, fade within three to four hours, and are dose-dependent [3].

Several incidences of non-arteritic anterior ischemic optic neuropathy (NAION) have been recorded in sildenafil users [5]. PDE5 inhibitors should not be used by patients taking nitrate compounds since they can cause abrupt hypotension. Visual problems such as color perception changes, blurred vision, and NAION were also reported in patients taking PDE5 inhibitors [6].

The onset of NAION is usually sudden and painless, and it is linked with any pattern of visual field reduction. Reduced visual acuity, color vision impairment, visual field abnormalities, or hemorrhages in the form of flames are all potential symptoms and signs. Pain and headache were experienced by a small percentage of patients (almost 10%) [6]. The pathophysiology of NAION, however, is still unknown. The most widely recognized theory is that NAION is caused by small artery diseases, such as short posterior ciliary artery occlusion, which supply the optic nerve head, resulting in optic nerve head ischemia and infarction [6]. Most visual symptoms can be reversed weeks after the medicine is stopped [1]. Even in patients with pre-existing vascular retinal disorders, PDE5 inhibitors do not appear to cause persistent visual loss [3]. Between one and 12 hours after using sildenafil, several patients developed NAION. Bilateral simultaneous NAION has been reported in several patients [7]. Up to three weeks after using sildenafil, visual abnormalities have been observed [8].

The risk of developing NAION is increased by several other factors; optic nerve head anomalies, such as drusens of the optic nerve head and a small cup-to-disc ratio or the absence of cupping, genetic components, underlying systemic risk factors, such as hypertension, nocturnal hypotension, dyslipidemia, diabetes mellitus (DM), hypercoagulable states, sleep apnea syndrome, and hypoperfusion, cataract surgery, and systemic medications, such as amiodarone [6]. This review article will discuss the risk of NAION associated with the use of PDE5 inhibitors.

Search strategy

Detailed research was performed using the following keywords: Non-Arteritic Anterior Ischemic Optic Neuropathy, NAION, PDE5 Inhibitors, sildenafil, erectile dysfunction, Sexual dysfunction, Adverse Effects, and Viagra, in order to select the studies analyzing and assessing the association of PDE5 inhibitors for ED on NAION. All the articles considered were chosen without the restriction of time of publication or study type, i.e., traditional reviews, systematic reviews, clinical trials, case-control, and cohort studies. Age and ethnicity-specific research were not conducted. The search did not include any demographic restrictions. The articles selected were all written in English. Data was collected from 2005 up to March 2022.

## Review

Erectile dysfunction

Mechanism/Pathophysiology

The maintenance of normalcy in sexuality is dependent on the coordination of the human multi-system, which consists of the nervous system, the cardiovascular system, the endocrine system, and the reproductive system. Some pathological mechanisms such as endothelial dysfunction, neurogenic factors, hormonal pathways, and hemodynamic factors could directly or indirectly affect male sexual dysfunction [9].

While the smooth muscles are contracted, the penis remains in its flaccid state. A combination of adrenergic (noradrenaline) control, intrinsic myogenic control, and endothelium-derived contracting factors (prostaglandin and endothelins) is responsible for the regulation of smooth muscle contraction. Erection occurs upon sexual stimulation and after nitric oxide (NO) is released from non-adrenergic non-cholinergic (NANC) nerve fibers, and acetylcholine is released from parasympathetic cholinergic nerve fibers; the result of the ensuing signaling pathways is increased cyclic guanosine monophosphate (cGMP) concentrations, decreased intracellular Ca^+2^ levels, and smooth muscle cell relaxation. Once the smooth muscles relax, blood fills the lacunar spaces in the corpora cavernosa, which leads to compression of the subtunical venules, thus resulting in the blockage of the venous outflow (veno-occlusion). As the cGMP is hydrolyzed by PDE5, the process is reversed. Any interruptions in these processes can result in ED [10]. Any endothelium damage by exposure to chronic disease and toxins and the vasculature can demonstrate decreased production, impaired release, and increased destruction of bioavailable NO, which can lead to a disruption of the homeostasis typically demonstrated by healthy erectile tissue [11].

Erectile Dysfunction Association

Once thought to be psychogenic in etiology, ED nowadays is associated mainly with vascular diseases [12]. Diabetes mellitus, hypertension, hyperlipidemia, obesity, testosterone deficiency, and prostate cancer treatment are some of the more common conditions associated with ED. Some psychological causes are performance anxiety and relationship issues. ED can also be aggravated or caused by medications and substance use; antidepressants and tobacco use are considered the most common [13]. 

Developing evidence suggests that the formation of atherosclerotic plaques in blood vessels, considered a major pathophysiological mechanism, which leads to the development of cardiovascular disease, is responsible for causing functional damage to the inner layer of the vascular wall, the endothelium [12].

Furthermore, it was observed that men with ED have a higher degree of insulin resistance, which may result in endothelial dysfunction in the corpus cavernosum, increased oxidative stress, reduced NO concentrations (which have a vasodilator effect), and increased endothelin-1 levels (a potent vasoconstrictor), all of which results in erectile mechanism [14]. Serving as a selective barrier of a blood vessel wall, endothelial cells, which act as a selective barrier, also secrete a wide variety of vasoactive substances, consisting of NO, endothelin-1, and thromboxane 2. When the endothelial integrity is impaired, its permeability is increased; thus, it activates platelets and leukocytes, resulting in the activation of various cytokines and reducing NO production. This leads to impaired vasodilation [12]. ED is considered an early marker of cardiovascular disease, additionally also associated with the increased possibility of cardiovascular disease. ED would precede a cardiovascular occurrence by two to five years [14].

Erectile Dysfunction Treatment

The treatment of organic ED currently consists of four common types of therapy: oral drug therapy, vacuum tumescence devices, intracavernous vasoactive drug injections, and penile prosthesis implantation. Some other therapies are inclusive of psychosexual counseling, sex therapy, lifestyle changes, and gene therapy [11]. First-line treatments for ED are oral PDE5 inhibitors. Alprostadil and vacuum devices are second-line [13]. While PDE5 inhibitors are commonly used as the first-line treatment for ED, alprostadil is mainly used for patients with severe ED who display resistance to PDE5 inhibitors, ED related to neuronal damage from diabetes, or non-nerve-sparing pelvic surgery, and individuals with contradictions of PDE5 inhibitors [15].

Phosphodiesterase type 5 inhibitors

When Pfizer gained the first FDA clearance for sildenafil (Viagra), the first accepted oral medication for ED, there was a lot of enthusiasm about it. Sildenafil was designed as an anti-hypertensive and antianginal drug because of its function as a vasodilator. However, given the absence of clinical effectiveness for these endpoints and the fact that erections are a frequent side effect, the company quickly decided to focus on ED as its main target. Vardenafil and tadalafil obtained FDA clearance in 2003, making three drugs in the same class available for the treatment of ED [16]. Within the general population, the introduction of PDE5 inhibitors has transformed ED treatment with an average success rate of 60-70% [17].

Mechanism of Action

Although the pharmacological components are very similar in nature, the modest changes in side effect profiles and the time of the therapeutic response after treatment caused by the PDE5 inhibitors differing chemical selectivity, bioavailability, and or metabolism can be explained [16]. As a selective PDE5 inhibitor, sildenafil can potentiate the downstream effects of nitric oxide on smooth muscle relaxation and vasodilation through its impact on the cGMP pathway in the corpora cavernosa, also in the pulmonary vasculature, and the retina [18]. Normal ocular blood flow regulation and photoreceptor transduction depend on the NO/cGMP system [4].

These drugs contribute by constraining the enzyme PDE5. It is also found in the vessels' smooth muscle cells. By constraining the enzymes, these medications also stop PDE5 from degrading cGMP. GMP has the ability to activate protein kinase G, which causes the vascular smooth muscle to relax. Preventing PDE5 from degrading cGMP can result in a buildup of cGMP in the vascular smooth muscle, which can dilate vessels by phosphorylating several downstream effector molecules [19]. 

PDE5 inhibitors work to raise cGMP levels, which may lead to vasodilation and reduced perfusion of the optic nerve [4]. An erection lasts longer when the penile arteries are dilated. Additionally, PDE5 inhibitors work to improve endothelial function whilst reducing the apoptosis of vascular smooth muscle cells in the corpus cavernosum [19]. Atrial natriuretic peptide, renal function, pulmonary vascular resistance, and retinal blood flow are all modulated by PDE5 [3]. Different types of PDE inhibitors and their generic names are described in Table 1 [20].

**Table 1:** Different types of phosphodiesterase inhibitors and their generic names Adapted from Padda and Tripp, StatPearls Publishing [20] PDE: phosphodiesterase

Type	Generic name
PDE-3 inhibitors	cilostazol, dipyridamole, milrinone, and amrinone
PDE-4 inhibitors	roflumilast, apremilast, crisaborole
PDE-5 inhibitors	sildenafil, tadalafil, vardenafil, and avanafil
Non-specific PDE inhibitors	theophylline, ibudilast

Systemic and Ocular Effects

They are categorized into 11 family units and are based on structural resemblance [3]. All PDE5 inhibitors work in the same way. They still have a different PDE isozyme selectivity with PDE6, though (sildenafil). Moreover, PDE11 (tadalafil) is impacted by cross-reactivity, which causes the manifestation of certain adverse effects related to the usage of PDE5 inhibitors [9]. PDE6 is an enzyme that is found in the retina and affects cGMP concentrations as well as visual signal transduction. It also stimulates the activity of the retinal pigment epithelial (RPE) pump, which helps the retina absorb the fluid underneath [3].

Headache, nasal congestion, flushing, and dyspepsia are a few of the frequent side effects of sildenafil that are frequently linked to its pharmacologic characteristics as a PDE5 inhibitor and as a mild PDE6 inhibitor (i.e., visual disturbances) [3].

Modifications in color and light perception, brief changes in electroretinogram, impaired eyesight (central haze, transitory decrease in vision), conjunctival hyperemia, ocular discomfort, and light sensitivity are among the most frequent ocular adverse effects [3]. There have also been reports of optic neuropathy (anterior and posterior ischemic optic neuropathy) and atrophy, angle-closure glaucoma, branch retinal artery occlusion, central serous retinopathy, and glaucoma [4]. However, even in patients with pre-existing vascular retinal diseases, PDE inhibitors do not seem to contribute to permanent vision damage [3]. While published literature suggests a possible connection between hepatotoxicity and sildenafil usage, the mechanism that motivates liver toxicity is still unidentified, which ranges from an asymptomatic elevation of liver enzymes to fulminant hepatic failure [17]. Table 2 lists the FDA-approved PDE5 inhibitors [20].

**Table 2:** List of the FDA-approved PDE inhibitors and their related medical indications. Adapted from Padda and Tripp, StatPearls Publishing [20] PDE: phosphodiesterase

Medical problem	FDA-approved medication
Erectile dysfunction (ED)	sildenafil, tadalafil, vardenafil, and avanafil
Benign prostatic hyperplasia (BPH)	tadalafil
Pulmonary arterial hypertension(PAH)	sildenafil, tadalafil
Psoriatic arthritis (PA)	apremilast
Psoriasis	apremilast
Chronic obstructive pulmonary disease (COPD)	roflumilast, theophylline
Peripheral arterial disease (PAD)	cilostazol, pentoxifylline
Postoperative thromboembolic prophylaxis	dipyridamole
Decompensated cardiac failure	milrinone, amrinone
Atopic dermatitis	crisaborole
Thrombocythemia	anagrelide
Neonatal apnea	caffeine citrate

Overall, the opposing event rate of tadalafil is equivalent to that of sildenafil. However, although the specific adverse events differ, compared to sildenafil, tadalafil is associated with a reduced prevalence of flushing and a higher incidence of myalgia and back discomfort. Thus, tadalafil may be a better option for the treatment of ED [21]. 

Implications of PDE5 Inhibitors on Chorioretinal Diseases

After ingestion of 50-100 mg of sildenafil within a three-hour period, an increase in perfusion and thickness of the choroid has been recorded by spectral-domain optical coherence tomography (SD-OCT) and swept-scan high-frequency digital ultrasound [3]. Sildenafil could have a significant vasodilatory effect, which can result in increased choroidal blood flow as the cause of a direct impact on the smooth muscle relaxation in the choroidal vessel walls [5]. The sildenafil-induced alterations in choroidal perfusion and PDE6 inhibition were related to the ocular manifestations [3]. 

RPE cells express the PDE2, PDE5, and PDE9 isoforms, which control the levels of cGMP intracellularly. The RPE pump appears to be activated by the cGMP of RPE cells, which aids in the absorption of subretinal fluid [3]. Since PDE5 inhibitors increase the perfusion and thickness of the choroid, it results in the engorgement of the choroidal vasculature. Some individuals may experience leakage across the RPE and accumulated subretinal fluid, which could lead to central serous retinopathy manifested with distorted or loss of central vision, decreased color perception, and relative scotoma [5]. Except for tadalafil, sildenafil, vardenafil, and avanafil all block PDE6 and gene mutations that result in faulty PDE6 enzymes, which cause high amounts of cGMP photoreceptor cell death (3).

Vascular Effects

PDE5 inhibitors work to increase cGMP concentrations, which may therefore cause vasodilation and a decrease in optic nerve blood supply. Hypoperfusion of the optic nerve may also result from the presence of PDE5 in the smooth muscle cells of the blood vessels all over the body. Furthermore, PDE5 inhibitors, because of the mild systemic vasodilator effects, might induce systemic hypotension [4]. The hypotensive influence of sildenafil is most likely associated with PDE3 inhibition, and the simultaneous use of nitrates is contraindicated [3].

NAION

Ischemic Optic Neuropathy Classification

Ischemic optic neuropathy ​​​​​is of two types: (a) anterior and (b) posterior ischemic optic neuropathy. Anterior ischemic optic neuropathy is further of two types: (a) arteritic anterior ischemic optic neuropathy (AION) and (b) NAION [22]. NAION is a cause of reduced vision leading to permanent loss of vision. It has also been reported very seldom postmarketing in its finite association with the use of PDE5 inhibitors, including Viagra. A significant portion of these patients had underlying anatomical and vascular risk factors predisposing NAION development; some of these risk factors include disc at risk (crowded disc), age over 50, DM, hypertension, coronary artery disease, hyperlipidemia, and smoking. It is not conceivable to decide whether these occasions are related straightforwardly to the utilization of PDE5 inhibitors, to the patient's fundamental vascular chance variables or anatomical defects, to a combination of these components, or to other components [23]. The NAION patients were found to be at an increased hazard for ischemic heart disease and ischemic stroke compared to patients without NAION [24].

Epidemiology of NAION

NAION is the foremost common ischemic optic neuropathy sort [24]. The prevalence of NAION within the United States has been evaluated to be anywhere between 2.3 to 10.2 per 100,000. In African Americans, it is less prevalent and is most common in Caucasians probably since African Americans are more likely to have a large physiologic cup and less likely to have a crowded disc, which is the leading predisposing factor for creating NAION [25]. NAION is a progressive disease in nature, and the rate of contralateral eye involvement is 15-20% in the following five years [26,27].

Risk Factors

NAION impacts elderly men more. Hypertension, DM, hyperlipidemia, stroke, and prothrombotic disorders were all reported as NAION-related cardiac and cerebrovascular diseases. Hyperhomocysteinemia, nocturnal hypotension, and other conditions [24,26]. Several other systemic and ocular factors have also been studied in previously published articles, including smoking, obstructive sleep apnea, PDE5 inhibitors, depression, anemia, chronic obstructive pulmonary disease, hypothyroidism, small optic disc, age-related macular degeneration, and glaucoma [26].

In numerous studies, arterial hypertension, DM, and atherosclerosis are established risk factors for NAION occurrence. Atherosclerosis contributes to both systemic vascular diseases and NAION formation. The atherogenic indices were significantly higher in NAION patients than in controls [24]. During a hypertensive crisis, a young, healthy patient presented sequential bilateral atypical NAION secondary to massive consumption of licorice root extract over a period of five years [28].

Pathophysiology

Even though PDE5 inhibitors mainly cause vasodilation and hypotension, their effects on NAION pathogenesis remain unclear [26]. There is no precise pathophysiology for NAION; however, there is a theory that localized edema occurs due to hypoperfusion of the optic nerve head. It is thought that in individuals prone to compartment syndrome (those with a crowded disc), when optic disc edema occurs, the swelling affects the neighboring axons leading to a compartment syndrome [25]. This results in significant swelling and ischemia of the axons, ganglion cell death, loss of function of the affected axons, secondary optic nerve damage, and demyelinating alterations in the optic nerve [25,29].

Diagnosis

The lack of confirmatory tests makes NAION diagnosis difficult, especially when there is bilateral sequential involvement [27]. It can be difficult to distinguish NAION from arteritic AION during the initial evaluation [30]. Severe loss of vision, giant cell arteritis (GCA) manifestations, and elevated inflammatory markers consistent with an inflammatory syndrome are all clear warning symptoms and signs that strongly support the diagnosis of arteritic AION [30]. Acute, painless unilateral vision loss is the most common symptom, which increases the risk of contralateral vision loss. With optic disc edema and an afferent pupillary defect, the condition can worsen over hours or days. In the visual field, altitudinal defects are a more common occurrence [1].

The macula is more vulnerable to ischemia and hypoxia in NAION due to the acute decrease in perfusion [29]. The papillomacular nerve fibers may not be involved at all, which explains why many eyes with NAION have normal VA. As a result, information on both VA and the entire peripheral visual field is required to assess visual outcomes in NAION [22]. Even though some of the affected eyes had good central vision, they had a tubular vision or visual island [29]. Visual impairment (hand motion or worse) is common in arteritic AION but uncommon in NAION [30]. The most common differential diagnosis in patients over 50 is GCA; thus, to rule out this potentially curable illness, inflammatory markers should be evaluated in all patients over 60 with optic disc edema [25]. 

Macular microperimetry may be a better method for assessing NAION damage [29]. NAION early optic disc edema may affect the optical coherence tomography (OCT) examination because it is a structural test. On the other hand, microperimetry is a functional examination that is better able to reflect visual function, and its results are not altered by the presence of edema [29]. A gadolinium-enhanced MRI of the orbits should be performed in certain cases to differentiate between NAION, and inflammatory optic neuropathy and most often will show enhancement of the optic nerve head in inflammatory neuropathies [25]. MRI findings, especially during the acute phase, can be non-specific [27]. 

NAION Presentation

Up to six months, VA and visual fields showed improvement or worsening, with no significant changes after that. At the initial visit, about half of the NAION eyes had almost normal VA (20/15 to 20/30). As a result, the presence of normal VA does not rule out the possibility of NAION. All eyes with classical NAION, on the other hand, have a visual field loss of varying severity [22]. Very poor VA has been reported in NAION patients, and according to numerous large studies, 4-14% of NAION patients have a very poor vision [30].

In the contralateral eye, all patients with very poor vision had a crowded disc, i.e. small cup to disc ratio [30]. When acuities range from 20/16 to 20/63, color loss generally correlates linearly with acuity loss. In the case of normal acuity, color perception is retained or nearly so. The degree of dyschromatopsia tends to be proportional to the reduction in acuity when acuity decreases [31].

Atypical NAION can have a more subtle clinical presentation than typical cases, with milder loss of visual acuity and a higher frequency of bilateral involvement. In addition, it has been linked to medications such as amiodarone, 5-alpha-reductase inhibitors, nasal decongestants, and epinephrine, as well as tumors, prothrombotic states, and perioperative bleeding [28]. Table 3 summarizes several previously published articles that have shown an association between PDE5 inhibitors and NAION [7,32-36].

**Table 3:** Studies that support the association between PDE5 inhibitors and NAION Table credit: first author, Hor M NAION: non-arteritic anterior ischemic optic neuropathy; PDE5: phosphodiesterase 5

Finding	Studies	Concise Methods	Brief Outcomes
	Galvez-Ruiz and Arishi, 2013 [32]	Case series	10 patients with mean age of 50.7 years who developed NAION after regular intake of sildenafil ( > 2-3 times per week) during the weeks and months presented in one eye
	Pomeranz and Bhavsar, 2005 [33]	Case series	Six patients aged 50-69 presented with vision loss within 36 hours after taking sildenafil of variable doses and one 58-year-old patient developed headache almost immediately after taking 50 mg sildenafil
	Moschos and Margetis, 2011 [7]	Case report	A 55-year-old male with simultaneous bilateral NAION eight months after continuous use of sildenafil 50 mg four to five times a month
NAION	Gruhn and Fledelius, 2005 [34]	Case report	A 69-year-old male developed NAION 18 hours after taking 50 mg sildenafil.
	Kim and Kim, 2012 [35]	Case report	A 54-year-old male developed a unilateral decreased visual field three days after two doses of udenafil 100mg 2 days apart.
	Tarantini et al., 2012 [36]	Case report	A 60-year-old male developed sudden bilateral decrease of vision 16 hours after thee consecutive 50 mg daily sildenafil

Treatment/Management

Despite the lack of definitive treatment for NAION, Hayreh et al. demonstrated that patients who were prescribed systemic steroids during the acute phase, when optic disc edema was still present, had improved VA and visual field compared to those who were not [37]. Treatment with steroids, on the other hand, did not seem to have a benefit on the vision, and prior antiplatelet therapy failed to prevent NAION from occurring, according to Dattilo et al. [30].

A study of optic nerve sheath decompression for the treatment of NAION found that it was "not an appropriate treatment for NAION and showed that it was harmful" [22]. Although topical brimonidine has been suggested to have neuroprotective properties, it has not been statistically proven to improve VA when compared to placebo [27]. 

Limitations

In this review article, there are numerous limitations, including the fact that the study groups were relatively small; thus, the results cannot be generalized. The search strategy for this article review was limited to two databases, PubMed and PubMed Central (PMC). Additionally, statistical analysis was not the primary purpose of the supporting point. This study does not provide any valid evidence that the use of Viagra may increase the risk of NAION. It was challenging to establish a substantial correlation between the use of PDE5 inhibitors and NAION since the latter had several confounding variables and shared certain contributing factors with ED. There were no included randomized controlled studies to support the causation.

## Conclusions

NAION has been reported rarely in men after taking sildenafil or other PDE5 inhibitors for ED. It is a rare ophthalmic disease with multiple contributing risk factors. Pathogenesis is unknown and there is no definitive treatment available. To prevent further attacks, it is crucial to identify and manage all systemic and local risk factors. There is no conclusive evidence of taking PDE5 inhibitors as a cause of NAION. Patients who are on PDE5 inhibitors, especially those with co-existing predisposing risk factors like DM, should be warned about the possibility of ischemic ocular side effects. It is important to conduct additional prospective randomized, double-blind placebo control studies with bigger sample sizes and comprehensive designs. An example would be a digital fluorescein angiography to compare the perfusion of the optic nerve, choroid, and retina before and after consumption of varying doses of sildenafil in one group and placebo in the other group. This study will further provide a solid correlation between the prevalence of NAION and PDE5 inhibitors. Clinicians should prescribe PDE5 inhibitors carefully after weighing their benefits against probable severe adverse events. Furthermore, physicians should document any visual symptoms observed during PDE5 inhibitors treatment and refer patients to ophthalmology for management and follow-up.
